# Interfering with sleep apnea

**DOI:** 10.1186/s42234-023-00139-w

**Published:** 2024-01-24

**Authors:** Nigel Paul Pedersen, Raul Castillo Astorga

**Affiliations:** 1 Department of Neurology, School of Medicine and Center for Neuroscience, University of California, Davis, 1515 Newton Court, Davis, CA 95618 USA; 2 Graduate Program in Biomedical Engineering, University of California, Davis, CA 95618 USA

**Keywords:** Temporal interference, Temporally-interferring electric fields, Sleep apnea, Non-invasive stimulation, Hypoglossal nerve, Hypopnea

## Abstract

The effects of electromagnetic interference have been hiding in plain sight for millennia and are now being applied to the non-invasive stimulation of deep tissues. In the article by Missey et al., the effect of non-invasive stimulation of the hypoglossal nerve by an interference envelope of interfering carrier waves is examined in mice and participants with sleep apnea. This stimulation is capable of activating the nerve and reducing apnea-hypopnea events. Temporally interfering electric fields have potential applications far beyond hypoglossal stimulation and may represent a revolutionary new approach to treating illness and understanding the functional organization of the nervous system.

In 1925, Ernest Merritt revealed the basis of ‘the brilliant iridescent colors exhibited by the wings of certain butterflies’ (Merritt 1925), describing the electromagnetic interference that liberates varied hues from what had been suspected to ‘structural colors’ since Robert Hooke’s *Micrographia (*Hooke 1665*)*. These observations are generalizable to other oscillatory waves, such as the beating pattern that we hear from an out-of-tune piano, radio telescope interferometry, through to oscillating electric fields.

The underlying effects of neural tissues vary markedly with stimulation frequency. A recent review proposes that stimulation ranging up to 500 Hz can have conventional physiological effects (Neudorfer et al. 2021). Still, we are only beginning to understand the effects of higher frequency electric fields. Generally, lower frequency stimulation (here < 500 Hz) activates voltage gated channels on neural elements - nodes of Ranvier, axon hillock, somata, and dendrites, thus exerting a wide range of physiologic effects. As one approaches the time course of channel conformational changes, around and above the kiolhertz range, effects are more variable and can include activation or inhibition. When the waveform is a sinusoid rather than a charge-balanced pulse (Lilly et al. 1955), impacts are less clear, but effects are generally less evident than those of low-frequency stimulation.

With the description of temporally-interfering electric fields by Grossman et al., this reduced or lack of effect of high frequency carriers and potential impact of the beating interference envelope was posited (Grossman et al. 2017). In this simple model, the overlapping field ‘envelope’ impacts neural tissues, while the high frequency ‘carrier’ frequencies do not. Unlike transcranial magnetic stimulation (tMS) and low-frequency alternative fields (transcranial alternating current stimulation, tACS) with rapid deacy of the fields from extracranial sources, this would enable the non-invasive targeting of deep tissues. While the TI carrier frequencies are also susceptible to this decay from the source by using transcranial temporal interference, the envelope can be focused on a deeper target. The deliberate use of this approach requires modeling that is non-trivial: Maxwell’s equations must be solved for the target, an accurate brain model (from participant imaging) may be required, and the orientation of the consequent envelop field may determine how this envelope interacts with somata, neuropil, and white matter (Mirzakhalili et al. 2020). Recent modeling work challenges the simple assumptions of temporal interference, and reveals more complex effects in the envelope that may be due to a combination of membrane rectification (channels that allow preferential direction of current flow in the membrane) and both resistive and capacitive filtering (Mirzakhalili et al. 2020). In these models, conduction block outside the envelope occurs, at least at the modeled carrier frequencies of 1 and 1.1 kHz. The authors argue that the clinical application of this method may be complex given stimulation at the envelope and suppression of neural activity outside of the envelope. Nonetheless, recent empirical results show great promise:, TI stimulation was recently used to focally modulate hippocampal activity and enhance the accuracy of episodic memories in healthy humans (Violante et al. 2023), validating both principle and effects of TI in humans. As another example of the successful use of TI, Missey et al. in this issue of *Bioelectronic Medicines shows* its usefulness in peripheral nerve stimulation for the treamtent of sleep apnea.

Sleep apnea is a highly prevalent sleep disorder that is associated with neurologic and cardiovascular morbidity (Yeo et al. 2023; Redline et al. 2023). One FDA-approved approach to treatment is hypoglossal nerve stimulation by a chronically implanted neurostimulator (Strollo et al. 2014). A recent randomized clinical trial of hypoglossal nerve stimulation, versus sham, in obstructive sleep apnea shows the efficacy of this approach across a broad range of patients, but without comparison to other treatments (Schwartz et al. 2023).

The study by Missey et al. examines the possibility of non-invasive temporal interference stimulation as an alternative to chronic device implantation. This study is premised on the efficacy of hypoglossal stimulation in obstructive sleep apnea, by increasing tongue muscle tone to help open the airway at the oropharynx, and employs the bilateral activation of the hypoglossal nerve by two sets of electrodes placed over the submandibular portion of the nerve. These pairs of four electrodes can generate two separate electric fields, each with their own carrier frequency. The carrier frequencies are set at 3 kHz, with a difference in carrier frequencies from 0.5 to 5 Hz and a modeled envelope amplitude of 1 mA. A 5 Hz envelope frequency is shown, in mice, to generate maximal compound muscle action potentials in the hypoglossal muscle, with accompanying strain gauge measurement of tongue movement force. A feasibility study is then conducted in twelve patients with obstructive sleep apnea. Four gel electrodes were placed along each side of the submandibular course of the hypoglossal nerves, and the amplitude of the modeled envelope fields was titrated until a tongue movement was visible while the participants were awake. TI stimulation was then applied during sleep, and the primary outcome of apnea-hypopnea index was compared to a recent prior sleep study. This uncontrolled single group human portion showed that all women (n = 4/4) and one man (n = 1/7) responded to the treatment, also showing that the applied current needed to be almost double in men versus women to show tongue movements (Missey et al. 2023). While these findings are of a preliminary nature, this non-invasive approach may be preferred or used to evaluate a patient’s response for subsequent device implantation.

While the best approach to the parameters, modeling and targeting of temporally interfering electric fields is still an active area of research, this study reveals the broad potential for this new technology. In our view, a determined effort is warranted to explore this approach to neurostimulation. While other non-invasive means of stimulation are in present use - focused ultrasound, transcranial magnetic stimulation, as well as transcranial direct and alternating current methods - this temporal interference approach, unlike the others, has the potential for more simple implementation, as well as the potential targeting of any regions within the nervous system. Apart from focused ultrasound, each of these methods primarily influences superficial tissues, and applying high currents in alternating current stimulation can activate nerves in the scalp and dura resulting in pain (Liu et al. 2018). Focused ultrasound is unlikely to be practical for ambulatory, bedside or chronic use, and it relies on transient or permanent lesions to nervous tissue (Krishna et al. 2018). Temporally-interfering fields are a special case of alternating current stimulation where the high frequency carriers have less effect on superficial tissues.

Further technical improvements are improving the targeting of TI. The exploration of multipolar methods affords more focal envelopes (Botzanowski et al. 2023), the optimization of orientation of TI can align the envelope with targeted axon tracts (Missey et al. 2021; Botzanowski et al. 2022), and the combination of this technique with organic substrate electrolytic photocapacitors can enable implanted transduces that are driven by light (Missey et al. 2022). An unresolved question is whether envelope field strengths can be high enough to replace conventional direct electrical brain stimulation, where current densities typically range up to 30 µC/cm^2^ (Drane et al. 2021). Applications are very broad, but we hope to see this technology used not only in a therapeutic context, but also to non-invasively explore the functional organization of the nervous system in humans. Applications to functional mapping prior to epilepsy surgery or treatment (Acerbo et al. 2022), cognitive neuroscience research, the treatment of movement disorders, and even the manipulation of sleep-wake network nodes in insomnia and coma are exciting potential applications.

## Data Availability

Not applicable.
